# Leveraging the iron-starvation response to promote ferroptosis

**DOI:** 10.18632/oncotarget.24395

**Published:** 2018-02-03

**Authors:** Samantha W. Alvarez, Richard Possemato

**Affiliations:** Richard Possemato: Department of Pathology, New York University School of Medicine, New York, New York, USA; Laura & Isaac Perlmutter Cancer Center, NYU School of Medicine, New York, New York, USA

**Keywords:** iron-sulfur cluster, ferroptosis, NFS1, cancer, oxygen

An important micronutrient, iron is used by the cell either as an ion or upon assembly into heme or iron-sulfur cluster (ISC) cofactors. Iron is generally not a free ion in the body, but is stored intracellularly by the protein ferritin and transported via association with transferrin. This iron sequestration is thought to occur because iron-mediated lipid peroxidation via Fenton chemistry can result from elevation in the free, or labile, iron pool. Cells in culture or in animals that experience iron overload can undergo cell death [1, 2], a process that is mitigated by glutathione peroxidase 4 (GPX4) [3], and recently termed “ferroptosis” [4]. An established mechanism for inducing ferroptosis involves inhibiting the cystine/glutamate antiporter xCT thereby depleting intracellular cysteine, which is used to generate the anti-oxidant glutathione (GSH) [5]. Without GSH, GPX4 fails to neutralize iron-mediated lipid peroxidation, leading to ferroptosis, a process recently shown to be important in cancer cells that exist in a mesenchymal and therapy resistant persister state [6]. Markers of ferroptosis have been established for cells growing in culture, but it is still unclear under what circumstances cells in an animal, including cancer cells in a tumor, are subjected to the oxidative stress necessary to undergo ferroptosis. While additional work is required to understand the relevance of this type of cell death to tumorigenesis, recent work suggest that ferroptosis can occur in the asthmatic lung, during renal failure, or upon brain trauma [7]. While inhibition of xCT can induce ferroptosis, simple depletion of GSH does not, suggesting that other downstream effects of blocking cysteine import are important for its induction. We recently arrived at model to explain this outstanding problem via an investigation into genes that are differentially required in the context of atmospheric (21%) and tissue level (3-5%) O_2_ [8].

Using RNAi-based loss-of-function screening in cell culture and tumor xenografts, we found that a significant number of shRNAs were differentially required when comparing survival in tumors versus cell culture, or at 21% oxygen versus 3% oxygen. These results led us to conclude that the difference in oxygen tension in tumor xenografts and cell culture is a major driver of differential essentiality. Two genes encoding critical steps of ISC biosynthesis, NFS1 and ABCB7, scored as top hits in both comparisons. NFS1 removes sulfur from cysteine for use in the synthesis of ISCs, cofactors which sensitive to damage by O_2_. ISCs are critical components for at least 48 proteins including DNA polymerases, components of the electron transport chain, and several metabolic enzymes.

**Figure 1:**
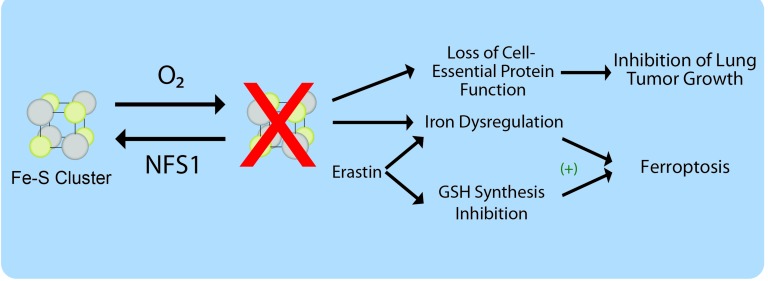
Model for involvement of NFS1 in lung tumor cell growth and ferroptosis NFS1 synthesizes iron-sulfur (Fe-S) clusters, which are sensitive to O_2_ degradation. Loss of clusters upon NFS1 inhibition hastens their depletion from cell essential proteins particularly in high O_2_ environments, limiting lung tumor formation. Additionally, NFS1 suppression can activate iron influx and release from intracellular stores, cooperating with glutathione (GSH) synthesis inhibition to promote cell death by ferroptosis.

While suppression of ISC biosynthesis inhibited cell proliferation at 21% O_2_ *in vitro*, growth at 3% O_2_, as orthotopic tumors in the mammary fat pad, or as subcutaneous xenografts was unaffected. Interestingly, breast cancer metastasis to the lung and primary lung tumor formation was significantly hindered by NFS1 knockdown. These observations led us to the conclusion that environmental oxygen concentration was driving the differential effects on tumor cell growth in the low oxygen environment of the mammary fat pad or subcutaneous space compared to the relatively higher oxygen environment of the lung. It is important to note that while established lung tumors are likely hypoxic, cells initially seeding lung metastases or pre-transformed lung airway epithelial cells may experience O_2_ concentrations near atmospheric levels. Consistent with this idea, analysis of human lung tumors revealed that NFS1 lies in a small genomic region undergoing positive selection in lung adenocarcinoma and NFS1 protein levels are elevated in well-differentiated tumors from this cancer type. These findings indicate that environmental oxygen levels experienced by incipient lung tumor cells can select for elevated NFS1 expression (Figure 1), and raise the possibility that there exist other situations in which environmental nutrient content can select for genetic alterations during tumorigenesis.

Having demonstrated a requirement for NFS1 specifically in elevated O_2_ conditions, we next sought to define the mechanism underlying this requirement. Because ISCs support the function of several cell-essential enzymes, cells must protect ISCs from oxidative damage to sustain proliferation. We demonstrated that in the intracellular environment, O_2_, but not other forms of reactive oxygen species, damage these clusters. In support of this finding, anti-oxidants fail to rescue the proliferation defects observed in NFS1 suppressed cells grown at atmospheric O_2_ levels, whereas culture at 3% oxygen readily rescues proliferation. Moreover, O_2_ affected ISC-dependent activities in NFS1 suppressed cells such as electron transport chain function and aconitase activity. Mechanistically, high O_2_ levels result in more rapid ISC turnover, and therefore suppression of NFS1 under these conditions leads to loss of ISCs from proteins with cell-essential functions.

In the course of performing these experiments, we noted that adding pro-oxidant molecules to cells in which NFS1 had been suppressed resulted in cell death despite these treatments not resulting in a measurable ISC defect. These results suggested that a downstream consequence of NFS1 suppression rendered cells susceptible to death by exogenous oxidants. Interestingly, an ISC cofactor present in the iron responsive protein IRP1 functions as an iron-response element binding protein when the ISC is lost or damaged, activating the iron starvation response [9]. Accordingly, suppression of NFS1 activated canonical iron starvation responsive proteins even at low O_2_, increasing the level of the transferrin receptor while repressing ferritin. Therefore, we concluded that the cell death observed upon NFS1 suppression and concomitant treatment with pro-oxidant molecules was the result of iron overloaded cancer cells undergoing ferroptosis. Suppression of NFS1 sensitized cells to both direct and indirect inhibition of GPX4 *in vitro* and to treatment with cyst(e)inase, an enzyme which can deplete serum cyst(e)ine levels thereby inducing oxidative stress, in a mouse xenograft model. Moreover, erastin treatment, which deprives cells of cysteine and therefore GSH produced from it, also results in a modest activation of the iron-starvation response. In accordance, NFS1 suppression strongly cooperates with inhibition of GSH biosynthesis to promote ferroptosis. These results are consistent with both ISC and GSH biosynthesis being downstream pathways inhibited by erastin and important for its induction of ferroptosis (Figure 1).

Based on these findings, NFS1 suppression offers two therapeutic avenues for targeting cancer. Suppression of NFS1 inhibits lung tumor growth as a result of loss of cell essential functions from cofactor depletion, and at the same time activates the iron starvation response, priming cells to undergo ferroptosis in response to oxidant treatment.
